# miR-431-5p regulates cell proliferation and apoptosis in fibroblast-like synoviocytes in rheumatoid arthritis by targeting XIAP

**DOI:** 10.1186/s13075-020-02328-3

**Published:** 2020-10-06

**Authors:** Yuejiao Wang, Kailin Zhang, Xiaowei Yuan, Neili Xu, Shuai Zhao, Linxin Hou, Lili Yang, Ning Zhang

**Affiliations:** 1 Department of Rheumatology and Immunology at Shengjing Hospital of China Medical University, Shenyang, Liaoning China; 2 China Medical University—The Queen’s University of Belfast Joint College, Shenyang, Liaoning China; 3 Department of Orthopedics at Shengjing Hospital of China Medical University, Shenyang, Liaoning China

**Keywords:** Rheumatoid arthritis, Synoviocytes, miR-431-5p, XIAP, Proliferation, Apoptosis

## Abstract

**Background:**

miR-431-5p is dysregulated in various cancers and plays an important function in the development of cancer. However, its role in fibroblast-like synoviocytes (FLSs) in patients with rheumatoid arthritis (RA) remains to be understood.

**Methods:**

Quantitative real-time polymerase chain reaction was used to detect the relative expression of miR-431-5p in synovial tissues and FLSs. Cell proliferation assays helped examine RA FLS proliferation. Flow cytometry was performed to determine apoptosis and cell cycle progression in RA FLSs. We used dual-luciferase assays to determine the correlation between miR-431-5p and its putative target, X-linked inhibitor of apoptosis (XIAP). Quantitative real-time PCR and western blotting were used to measure XIAP levels in synovial tissues and transfected RA FLSs.

**Results:**

miR-431-5p was downregulated in synovial tissues and FLSs of patients with RA. Upregulation of miR-431-5p prohibited cell proliferation and the G0/G1-to-S phase transition but promoted apoptosis in RA FLSs, while miR-431-5p inhibition showed the opposite results. miR-431-5p directly targeted XIAP in RA FLSs and reversely correlated with XIAP levels in synovial tissues. Notably, XIAP silencing partially restored the effects of miR-431-5p inhibition in RA FLSs.

**Conclusion:**

miR-431-5p regulates cell proliferation, apoptosis, and cell cycle of RA FLSs by targeting XIAP, suggesting its potential in the treatment of RA.

## Background

Rheumatoid arthritis (RA) is a chronic inflammatory autoimmune disease with a global prevalence of 0.5–1.0% [1]. Patients with RA may develop clinical hallmarks of joint swelling, arthralgia, and stiffness in the morning; without medical intervention, these symptoms aggravate as disease progresses [2]. Although there has been significant advancement in the treatment regimens for RA, patients continue to experience progressive articular damage with time (detectable in radiographs) and have high rates of articular deformity and other complications such as interstitial lung disease and cardiovascular diseases [3, 4]. Therefore, it is imperative to gain a comprehensive understanding of the pathogenesis of RA to develop effective preventive and therapeutic strategies.

Fibroblast-like synoviocytes (FLSs) constitute a major portion of the synovial intima and are pivotal to the development of RA. A healthy synovium is comprised of a superficial synovial lining named intima and a deeper zone called sub-lining or sub-intima. The intima is 2–3 cells thick in healthy individuals and 70–80% is FLSs [5]. However, RA patients possess hyperactivated FLSs that have tumor cell-like properties, including excessive proliferation with repressed apoptosis, migration, invasion, and persistent production of various inflammatory cytokines, chemokines, and matrix metalloproteinases [6]. These hallmarks contribute to the thickening of the synovium and formation of pannus, thereby culminating in articular deformity. However, there are no currently available drugs that target hyperactivated FLSs as treatment for RA.

MicroRNAs (miRNAs) are short 22-nucleotide transcript that are expressed in multiple organs and tissues [7] and involved in the pathogenesis of RA by modulating lymphocyte differentiation [8], bone homeostasis [9, 10], angiogenesis [11], and other properties of FLSs [10, 12–14]. We have shown that miRNAs are important for FLSs proliferation, apoptosis, and inflammation in individuals with RA [15, 16]. Studies have recently demonstrated that miR-431-5p is dysregulated in various human cancers, such as lung, liver, colon cancer, and squamous cell carcinoma [17–20]. However, the role of miR-431-5p in RA remains to be understood. We hypothesized that miR-431-5p is dysregulated in RA FLSs.

In this study, we have demonstrated that miR-431-5p was downregulated in RA FLSs and targeted the X-linked inhibitor of apoptosis protein (XIAP) to regulate cell proliferation, apoptosis, and cell cycle. These findings will help develop and test novel strategies for treating RA.

## Methods

### Patient samples

Synovial tissues of patients with RA (8 females) and normal synovial tissues of patients (6 females) receiving emergent traumatic amputation as healthy controls were collected from the Department of Orthopedics at Shengjing Hospital of China Medical University. All the patients met the 2010 ACR/EULAR classification criteria for RA [21] and were newly diagnosed without any DMARDs use. The clinical characteristics of RA patients were shown in Supplementary Table 1. Written informed consent was obtained from all the patients. All the experimental protocols used in this study were performed with the approval of the Ethics Committee of Shengjing Hospital of China Medical University.

### Cell culture and transfection

Immortalized FLS cell lines rather than primary FLS, healthy human fibroblast-like synoviocytes (HFLS), and human fibroblast-like synoviocytes from patients with RA (HFLS-RA) were commercially obtained from the Jennio Biotech Co., Ltd. (Guangzhou, China). HFLS and HFLS-RA cells were cultured in minimum essential medium (Corning, USA) and Dulbecco’s modified Eagle medium (Corning), respectively, supplemented with 10% fetal bovine serum (Gibco, USA), penicillin (100 U/mL), and streptomycin (100 mg/mL; Hyclone, USA). TNF-α (10 ng/mL) was used to stimulate HFLS-RA cells. The only one cell line, HFLS-RA, was used to perform follow-up functional assays.

miR-431-5p mimics, inhibitor, mimics and inhibitor negative control (NC), miR-410-3p mimics, and siRNAs against XIAP were synthesized by GenePharma (Suzhou, China). The sequences were as follows: miR-431-5p mimics: 5′-UGUCUUGCAGGCCGUCAUGCACAUGACGGCCUGCAAGACAUU-3′; miR-431-5p inhibitor: 5′-UGCAUGACGGCCUGCAAGACA-3′; miR-410-3p mimics: 5′-AAUAUAACACAGAUGGCCUGUAGGCCAUCUGUGUUAUAUUUU-3′; mimics NC: 5′-UUCUCCGAACGUGUCACGUTT-3′ (sense) and 5′-ACGUGACACGUUCGGAGAATT-3′ (antisense); inhibitor NC: 5′-CAGUACUUUUGUGUAGUACAA-3′; XIAP siRNA#1: 5′-GGUCAGUACAAAGUUGAAATTUUUCAACUUUGUACUGACCTT-3′; XIAP siRNA#2: 5′-GCAGGUUGUAGAUAUAUCATTUGAUAUAUCUACAACCUGCTT-3′; XIAP siRNA#3: 5′-CAUGGAUAUACUCAGUUAATTUUAACUGAGUAUAUCCAUGTT-3′. HFLS-RA cells were transfected with the 50 nM of the siRNAs using Lipofectamine 3000 (Invitrogen, USA) according to the manufacturers’ instructions.

### Cell counting kit (CCK)-8 assay

HFLS-RA cells were first seeded into five 96-well plates at a density of 5 × 10^3^ cells/well with 60–70% confluence. We added 100 μL of fresh medium supplemented with 10 μL of the CCK-8 reagent (Promega, USA) into each well before and after transfection for 24 h, 48 h, 72 h, and 96 h. We measured OD_490_ using a microplate reader (BioTke, USA) after 4 h of incubation.

### 5-Ethynyl-2′-deoxyuridine (EdU) assay

HFLS-RA cells were seeded into a 96-well plate at a density of 5 × 10^3^ cells/well with 60–70% confluence. After transfection for 30 h, the old media were replaced with 100 μL of fresh media supplemented with 50 μM EdU reagent (Ribobio, China). After incubation for 18 h at 37 °C, HFLS-RA cells were fixed using 4% paraformaldehyde and sequential treatment of Triton X-100 (Beyotime, China), Apollo mix, and DAPI. We acquired images from five random fields using a fluorescence microscope.

### Apoptosis assay

HFLS-RA cells were seeded into 6-well plates at a density of 5–10 × 10^4^ cells/well with 70% confluence. These cells were collected after 48 h of transfection. After incubation with Annexin V-FITC and PI staining reagents (Dojindo, Japan) at room temperature for 15 min (protected from light), we measured the rates of apoptosis using the FACSAria flow cytometer (BD, USA).

### Cell cycle assay

HFLS-RA cells were seeded in 6-well plates at a density of 5–10 × 10^4^ cells/well with 70% confluence. After transfection for 48 h, HFLS-RA cells were collected and fixed with 75% ethanol at − 20 °C for at least 2 h. After incubation with the PI staining buffer (Promega, USA) at room temperature for 15 min (protected from light), we determined the number of cells in the different phases using the FACSAria flow cytometer (BD).

### Luciferase assay

Wild-type (WT) and mutant sequences of the 3′ untranslated region (UTR) of XIAP with the putative miR-431-5p binding sites were synthesized and cloned into the pmirGLO vector (Promega). HFLS-RA cells were co-transfected with miR-431-5p mimics/mimics NC and WT/mutant constructs for 48 h and subjected to the dual-luciferase reporter assay (Promega) according to the manufacturers’ instructions.

### Quantitative real-time polymerase chain reaction (qRT-PCR)

Total RNA was isolated from synovial tissues and HFLS-RA cells using RNAiso Plus (Takara, Japan). We reverse transcribed 1 μg of RNA using the Mir-X™ miRNA First-Strand Synthesis kit and PrimeScript™ reagent kits (Takara) for the miRNA and mRNA samples, respectively. Gene expression was measured using the SYBR® qRT-PCR kit (Takara) and normalized to that of GAPDH (mRNA) or U6 (miRNA). Relative expression of genes was calculated using the 2^−△△Ct^ method. The primers using for PCR were as follows: 5′-TGTCTTGCAGGCCGTCATGC − 3′ (forward) for hsa-miR-431-5p; 5′-ACACACTTCGGGTTTCACGA-3′ (forward) and 5′-AAGTCCCTTCGTCTCCCTCA-3′ (reverse) for XIAP; 5′-ATGTTGCAACCGGGAAGGAA-3′ (forward) and 5′-AGGAAAAGCATCACCCGGAG-3′ (reverse) for GAPDH.

### Western blotting

Synovial tissues and FLSs were lysed using RIPA buffer (Beyotime). Total protein levels were measured using a BCA kit (Beyotime). Thereafter, 40 μg of protein was separated using 10% SDS-PAGE and electro-blotted onto PVDF membranes. The membranes were blocked using 5% non-fat milk, incubated with primary antibodies against XIAP (1:500), YY1 (1:500), and GAPDH (1:5000; Proteintech, China), and incubated with secondary antibodies (Proteintech). Protein bands were analyzed using Image J and normalized to GAPDH levels.

### Statistical analysis

All experiments were performed independently in triplicates. We used Student’s *t* test and one-way analysis of variance followed by Dunn’s multiple comparisons post hoc test to determine significance between differences in two or multiple groups, respectively. Data from the cell proliferation assay were compared using two-way analysis of variance. GraphPad Prism (v.7.0, CA) was used to analyze the data. *P* < 0.05 was considered statistically significant.

## Results

### miR-431-5p was downregulated in RA

**Fig. 1:**
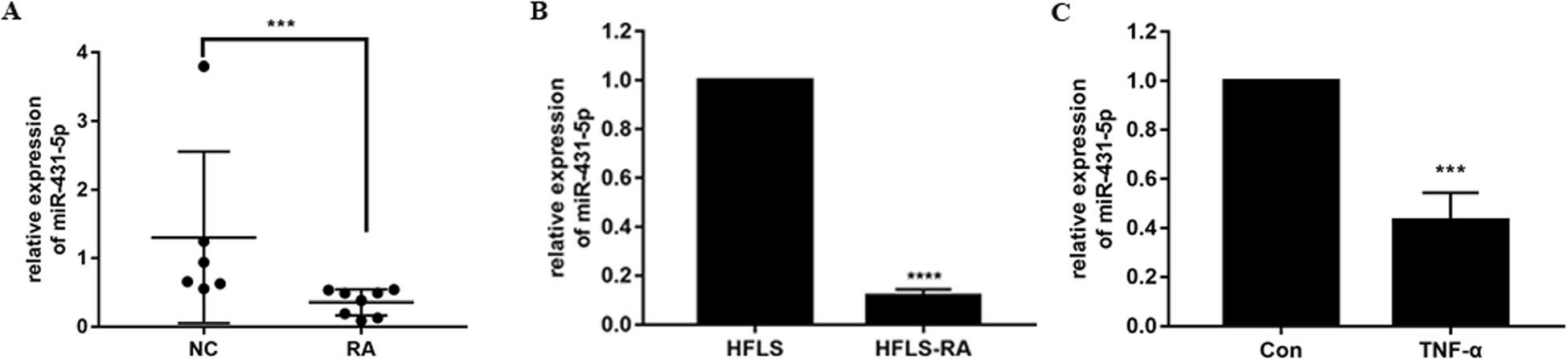
miR-431-5p was downregulated in RA. **a** Relative expression of miR-431-5p in synovial tissues of patients with RA (*n* = 8) and healthy controls (*n* = 6) by qRT-PCR. **b** Relative expression of miR-431-5p in HFLS and HFLS-RA cells by qRT-PCR. **c** Relative expression of miR-431-5p in HFLS-RA cells with or without TNF-α treatment by qRT-PCR. Each experiment was performed independently in triplicates. ***P < 0.001, ****P < 0.0001, compared with the NC groups

qRT-PCR showed a downregulation of miR-431-5p in synovial tissues from patients with RA as compared to that in the healthy cohort (*p* = 0.0007, Fig. 1a). Furthermore, we detected miR-431-5p levels in immortalized cell lines and HFLS and HFLS-RA cells. Accordingly, miR-431-5p was downregulated in HFLS-RA cells compared to that in HFLS cells (*p* < 0.0001, Fig. 1b). Moreover, miR-431-5p was reduced in HFLS-RA cells with TNF-α treatment compared with that without TNF-α treatment (*p* = 0.001, Fig. 1c), suggesting that dysregulated miR-431-5p might be involved in the development of RA.

### Overexpression of miR-431-5p suppressed cell proliferation in RA FLSs

**Fig. 2:**
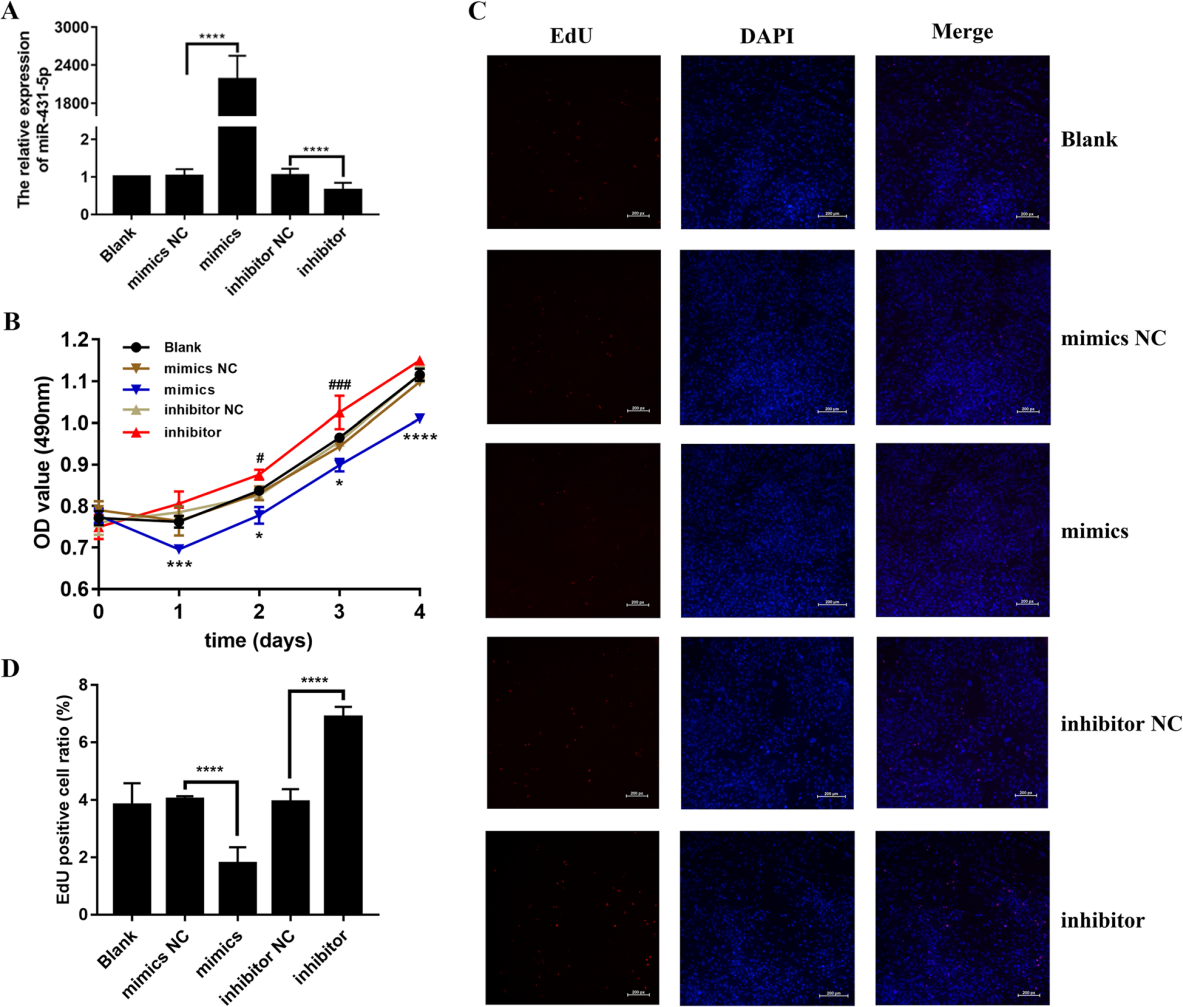
Overexpression of miR-431-5p suppressed cell proliferation in RA FLSs. **a** qRT-PCR for the relative expression of miR-431-5p in HFLS-RA cells transfected with miR-431-5p mimics or inhibitor. **b** Cell proliferation pattern of HFLS-RA cells transfected with miR-431-5p mimics or inhibitor for 0 h, 24 h, 48 h, 72 h, and 96 h using the CCK-8 assay. **c**, **d** Cell proliferation of HFLS-RA cells transfected with miR-431-5p mimics or inhibitor for 48 h by EdU staining assay. Each experiment was performed independently in triplicates. *P < 0.05, ***P < 0.001, ****P < 0.0001, compared with the NC groups

To elucidate the effects of miR-431-5p on cell proliferation in RA FLSs, we used the miR-431-5p mimics and inhibitor in HFLS-RA cells. The transfected HFLS-RA cells showed a ~ 2000-fold enhancement in miR-431-5p levels (*p* < 0.0001, Fig. 2a); HFLS-RA cells transfected with the inhibitor showed > 2-fold reduction in the levels of miR-431-5p (*p* < 0.0001). The NC samples showed no difference in miR-431-5p levels.

CCK-8 assays showed decreased proliferation in miR-431-5p-overepxressing HFLS-RA cells (*p* < 0.05, Fig. 2b). However, miR-431-5p inhibition significantly increased cell proliferation (*p* < 0.05, Fig. 2b). EdU staining assays were consistent with CCK-8 assay; miRNA mimics-transfected cells showed a decrease in the number of EdU-positive cells (*p* < 0.0001, Fig. 2c, d), while inhibition of miR-431-5p increased the number of EdU-positive cells compared to that of the control subsets (*p* < 0.0001, Fig. 2c, d). This suggests that upregulation of miR-431-5p suppresses HFLS-RA cell proliferation.

### Overexpression of miR-431-5p induced apoptosis and suppressed G0/G1-to-S phase transition in RA FLSs

**Fig. 3:**
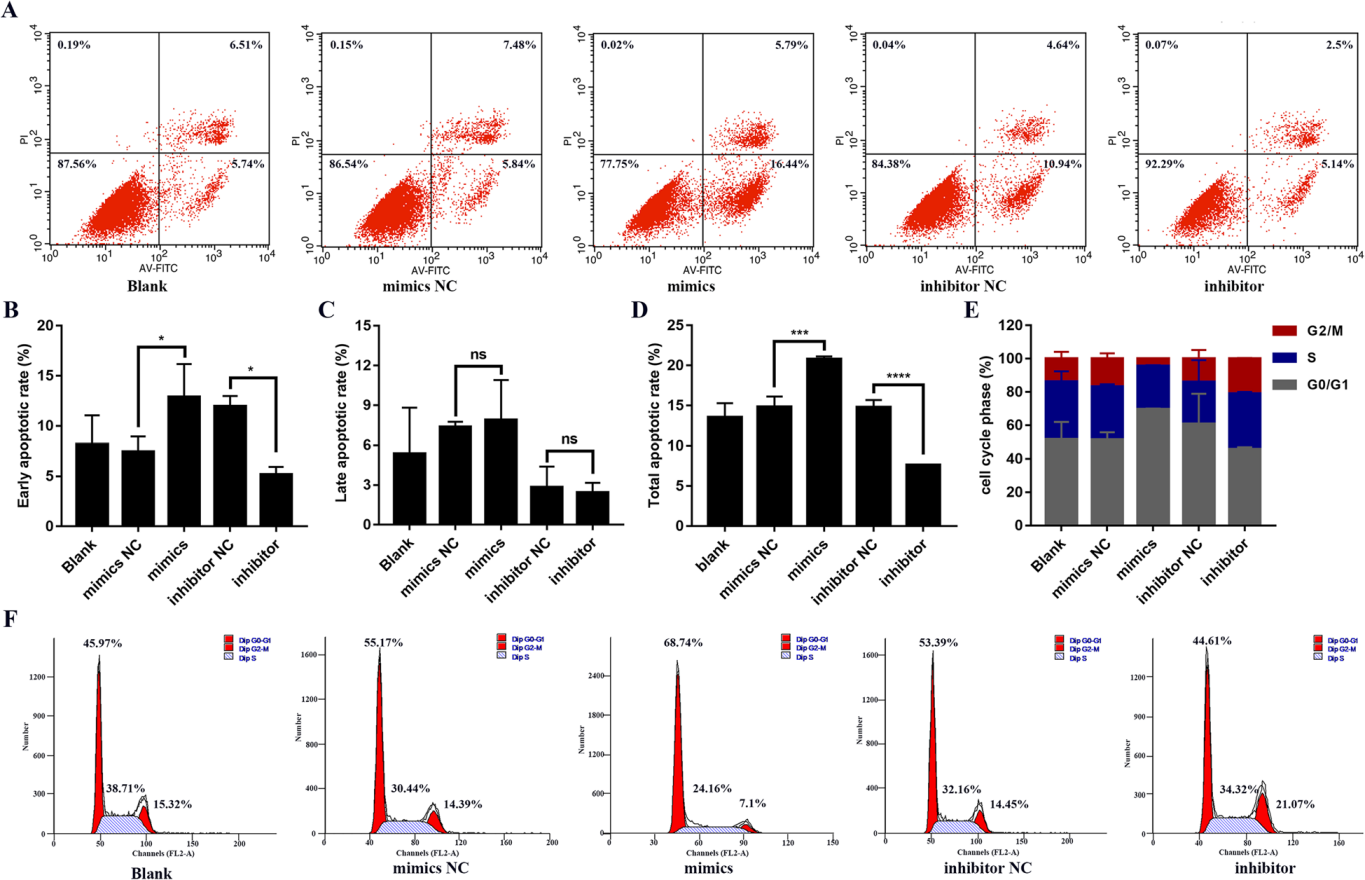
Overexpression of miR-431-5p induced apoptosis and suppressed G0/G1-to-S phase transition in RA FLSs. **a** Flow cytometry of Annexin V-FITC and PI-stained HFLS-RA cells transfected with miR-431-5p mimics or inhibitor for 48 h to determine cellular apoptosis. **b**–**d** Quantification of early (**b**), late (**c**), and total (**d**) apoptotic ratios of HFLS-RA cells transfected with miR-431-5p mimics or inhibitor. **e**, **f** Flow cytometry and quantification for cell cycle progression using PI-stained HFLS-RA cells transfected with miR-431-5p mimics or inhibitor for 48 h. Each experiment was performed independently in triplicates. *P < 0.05, ***P < 0.001, ****P < 0.0001, compared with the NC groups

We used flow cytometry to understand the role of miR-431-5p on apoptosis and cell cycle progression. As shown in Fig. 3a, miR-431-5p overexpression significantly enhanced apoptosis in HFLS-RA cells (*p* = 0.0004, Fig. 3d), particularly during the early phase of apoptosis (*p* < 0.05, Fig. 3b). Inhibiting miR-431-5p expression suppressed early and end-phase apoptosis in HFLS-RA cells (*p* = 0.0201 and *p* < 0.0001, respectively). However, apoptotic ratios showed no difference among five groups in the late phase of apoptosis in HFLS-RA cells.

Further, we explored the function of miR-431-5p on cell cycle progression in HFLS-RA cells. Flow cytometry showed that the ratio of G0/G1 phase HFLS-RA cells was significantly higher in cells transfected with the miR-431-5p mimics (*p* = 0.0253, Fig. 3e, f), while the ratio of G0/G1 phase HFLS-RA cells was lower in cells depleted of miR-431-5p as compared to that in their respective control subsets (*p* < 0.05). Thus, miR-431-5p may inhibit G0/G1-to-S phase transition in HFLS-RA cells. Conclusively, overexpression of miR-431-5p might suppress cell proliferation through inducing apoptosis and the G0/G1-to-S phase transition in RA FLSs.

### miR-431-5p directly bound XIAP in RA FLSs

**Fig. 4:**
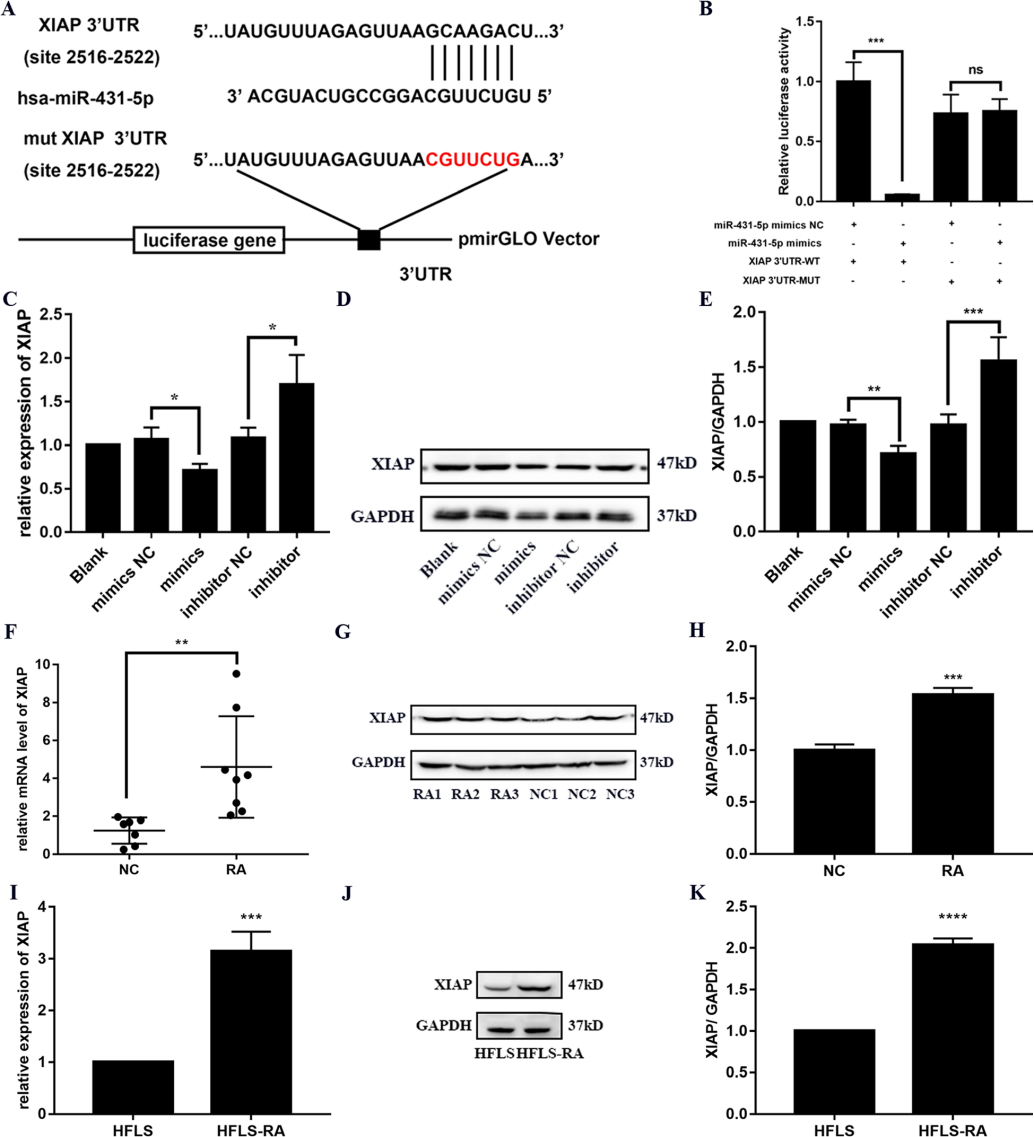
miR-431-5p directly bound XIAP in RA FLSs. **a** Binding between miR-431-5p and XIAP predicted by TargetScan. **b** Dual-luciferase reporter assay showing binding between miR-431-5p and the 3′ UTR of XIAP. **c** qRT-PCR for the relative expression of XIAP in HFLS-RA cells transfected with miR-431-5p mimics or inhibitor. **d**, **e** Western blotting to determine the levels of XIAP in HFLS-RA cells transfected with miR-431-5p mimics or inhibitor. **f** qRT-PCR for the relative expression of XIAP in synovial tissues of patients with RA (*n* = 8) and healthy controls (*n* = 6). **g**, **h** Western blots showing the levels of XIAP in synovial tissues of patients with RA (*n* = 3) and healthy controls (*n* = 3). **i** qRT-PCR for the relative expression of XIAP in HFLS and HFLS-RA cells. **j**, **k** Western blots showing the levels of XIAP in HFLS and HFLS-RA cells. Each experiment was performed independently in triplicates. *P < 0.05, **P < 0.01, ***P < 0.001, compared with the NC groups

The putative binding between miR-431-5p and XIAP was predicted by TargetScan (Fig. 4a). As shown in Fig. 4b, the miR-431-5p mimics reduced luciferase activity when co-transfected with the construct containing the WT 3′ UTR of XIAP (*p* = 0.0005). However, we observed no difference in luciferase activity in cells co-transfected with the construct containing XIAP 3′ UTR mutant, indicating binding between miR-431-5p and XIAP in HFLS-RA cells.

qRT-PCR and western blotting showed that miR-431-5p mimics significantly reduced the mRNA and protein levels of XIAP (*p* = 0.018 and *p* = 0.0069, respectively, Fig. 4c–e), while XIAP levels were induced in HFLS-RA cells after transfection with the inhibitor (*p* = 0.0108 and *p* = 0.0007, respectively). These results confirmed the interaction between miR-431-5p and XIAP in RA FLSs.

To further explore the miR-431-5p/XIAP signaling in RA, we determined the levels of XIAP in synovial tissues and cells. As shown in Fig. 4f–h, the mRNA and protein levels of XIAP were higher in synovial tissues of patients with RA as compared to that in the healthy cohort (*p* = 0.0069 and *p* = 0.0004, respectively). Consistently, the mRNA and protein levels of XIAP were upregulated in HFLS-RA cells as compared to that in HFLS cells (*p* = 0.0006 and *p* = 0.0001, respectively). Taken together, miR-431-5p may contribute to the development of RA by regulating XIAP.

Our previous studies have shown another miRNA, miR-410-3p, regulates cell proliferation, apoptosis, and cell cycle by directly targeting YinYang 1 in RA FLSs [16]. Since miR-431-5p shared overlapping effects with miR-410-3p in RA FLSs, we explored whether miR-431-5p and miR-410-3p also share similar mechanisms. As shown in Supplementary Fig 1 A-C, there were no significant differences of YY1 levels in HFLS-RA cells after transfection with miR-431-5p mimics, inhibitor and their respective NCs (all *p* > 0.05). However, XIAP levels were significantly inhibited in HFLS-RA cells after transfection with miR-410-3p mimics (*p* = 0.0006 and *p* = 0.0001, respectively, Supplementary Fig 2 A-C), suggesting that miR-431-5p and miR-410-3p might exert similar effects in RA FLSs through overlapping mechanisms.

### miR-431-5p regulated cell proliferation, apoptosis, and cell cycle progression via XIAP in RA FLSs

**Fig. 5:**
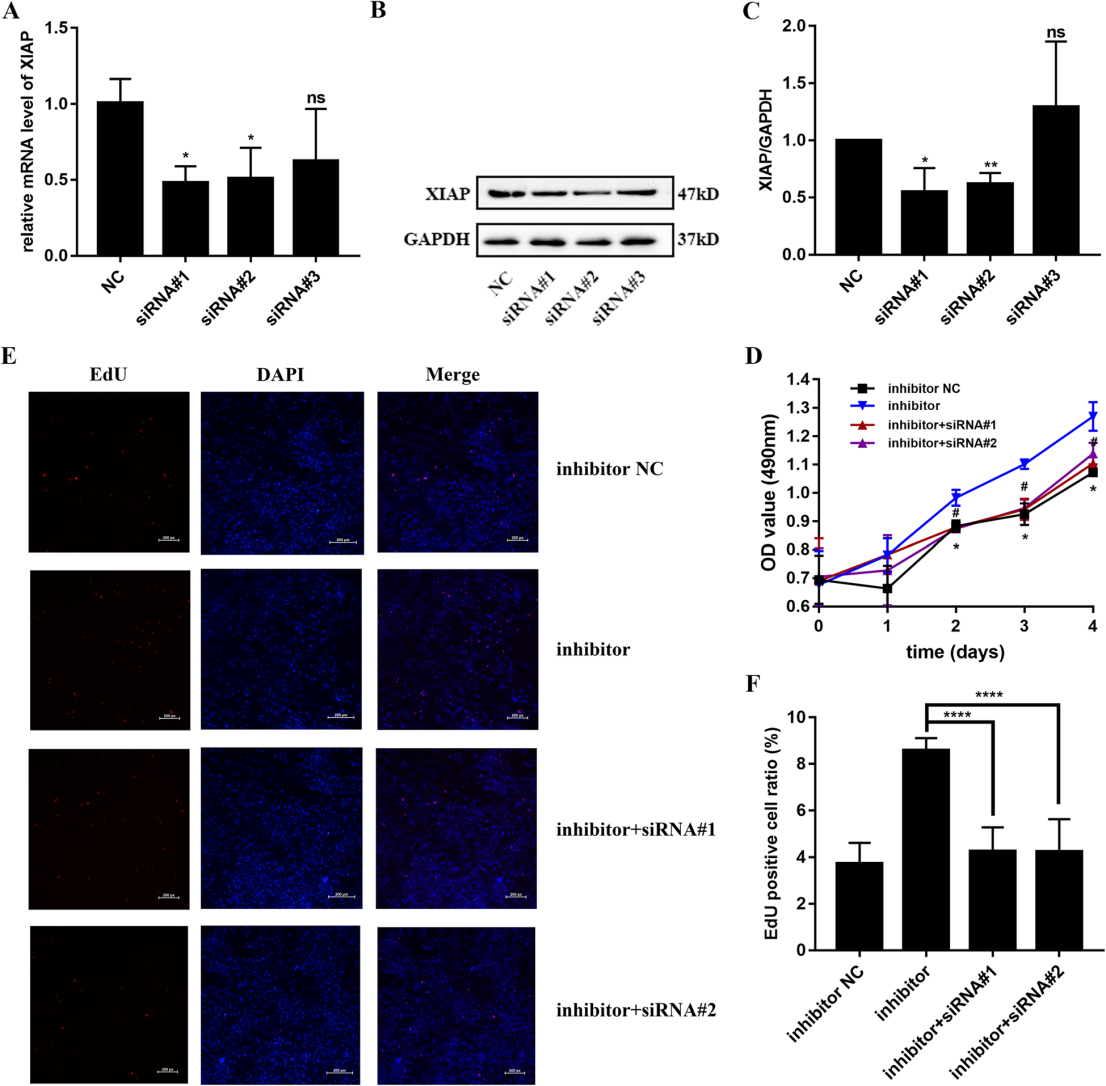
miR-431-5p regulated cell proliferation via XIAP in RA FLSs. **a** qRT-PCR for the relative expression of XIAP in HFLS-RA cells transfected with three siRNAs targeting XIAP. **b**, **c** Western blotting to determine the levels of XIAP in HFLS-RA cells transfected with three siRNAs against XIAP. **d** Proliferation of HFLS-RA cells transfected with miR-431-5p inhibitor and/or siRNAs against XIAP for 0 h, 24 h, 48 h, 72 h, and 96 h using CCK-8 assay. Asterisk for siRNA#1 and pound sign for siRNA#2. **e**, **f** Proliferation of HFLS-RA cells transfected with miR-431-5p inhibitor and/or siRNAs against XIAP for 48 h using EdU staining assay. Each experiment was performed independently in triplicates. *P < 0.05, ****P < 0.0001, compared with the NC groups

To understand the mechanism employed by miR-431-5p in regulating cell proliferation, apoptosis, and cell cycle progression in RA FLSs, we manipulated the expression of XIAP using siRNAs in HFLS-RA cells. As shown in Fig. 5a–c, siRNAs against XIAP reduced the mRNA and protein levels of XIAP in HFLS-RA (*p* < 0.05, *p* < 0.05, and *p* > 0.05, respectively). Since the siRNAs showed varied efficiency, we selected siRNA#1 and siRNA#2 for our subsequent functional assays.

CCK-8 assay showed that promotion of cell proliferation mediated by miR-431-5p inhibition was partially restored by XIAP silencing (all *p* < 0.05, Fig. 5d), particularly at 48 h, 72 h, and 96 h. Consistently, EdU staining indicated that the population of EdU-positive cells was lower in the cells co-transfected with the miRNA inhibitor and siRNAs against XIAP as compared to that in the cells transfected with the miRNA inhibitor (both *p* < 0.0001, Fig. 5e, f). Furthermore, inhibition of apoptosis induced by miR-431-5p inhibitor was restored by XIAP silencing (*p* < 0.0001 and *p* = 0.0001, respectively). Moreover, flow cytometry showed that the ratio of G0/G1 phase HFLS-RA cells was higher in cells co-transfected with the miRNA inhibitor and siRNAs against XIAP as compared to that in cells only transfected with the inhibitor (*p* = 0.0084 and *p* = 0.0068, respectively). This suggests that the increase in G0/G1-to-S phase transition induced by the miR-431-5p inhibitor was partially reduced by XIAP siRNAs. Thus, miR-431-5p suppressed cell proliferation and G0/G1-to-S phase transition and promoted apoptosis by targeting XIAP in RA FLSs.

## Discussion

RA is one of the most common autoimmune diseases in humans. However, its pathogenesis remains unknown. Research has shown the involvement of epigenetics in the pathogenesis of autoimmune diseases, especially RA [22]. miRNAs have recently been implicated in the development of RA. Targeting miRNAs have resulted in promising outcomes in animal models for RA [23, 24]. In this study, we have demonstrated that miR-431-5p is downregulated in synovial tissues and FLSs from patients with RA, indicating its potential role in the development of RA.

miR-431-5p is associated with the development of various cancers, such as lung cancer, glioblastoma multiforme, colon cancer, and hepatocellular carcinoma [17–19, 25]. miR-431-5p modulated cell proliferation, apoptosis, autophagy, migration, invasion, and angiogenesis. Upregulation of miR-431-5p prohibits cell proliferation and invasion via ATG3 in colon cancer [19]. Furthermore, miR-431-5p suppresses invasion by targeting UROC28 in hepatocellular carcinoma [18]. Consistent with previous studies, we observed that miR-431-5p overexpression suppresses cell proliferation and G0/G1-to-S phase transition but promoted apoptosis in RA FLSs, suggesting the potential of miR-431-5p in targeting excessively proliferative FLSs in patients with RA.

**Fig. 6:**
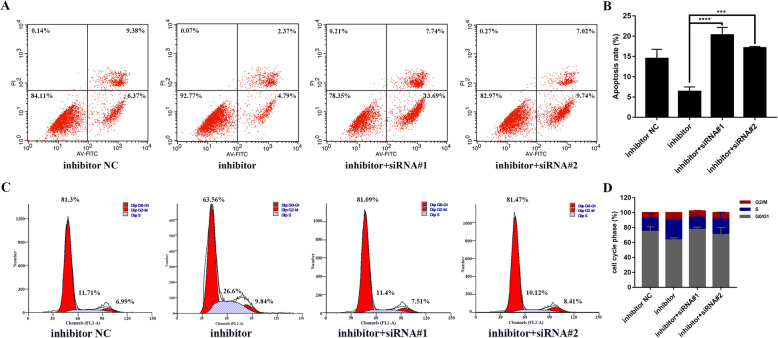
miR-431-5p regulated apoptosis and cell cycle progression via XIAP in RA FLSs. **a**, **b** Flow cytometry and quantification for total apoptosis using Annexin V-FITC and PI-stained HFLS-RA cells transfected with miR-431-5p inhibitor and/or siRNAs against XIAP for 48 h. **c**, **d** Flow cytometry and quantification of cell cycle progression using PI-stained HFLS-RA cells transfected with miR-431-5p inhibitor and/or siRNAs against XIAP for 48 h. Each experiment was performed independently in triplicates. **P < 0.01, ***P < 0.001, ****P < 0.0001, compared with the NC groups

Among the targets of miR-431-5p predicted by TargetScan, we focused on XIAP. XIAP is an inhibitor of apoptosis that selectively binds to and inhibits caspases [26]. Besides its well-known inhibitory role in apoptosis [27], Yu et al. reported the overexpression of XIAP in bladder cancer; XIAP also promotes lung metastasis in vivo [28]. Interestingly, XIAP confers resistance to cancer therapy and cell survival [29, 30]. Notably, XIAP inhibitors have shown promising outcomes in cancer therapy, including acute myeloid leukemia [31]. Dharmapatni et al. and Niederer et al. reported that XIAP was upregulated in synovium of RA [32, 33]. Particularly, Niederer’s group confirmed that XIAP was abundantly expressed throughout the entire synovial tissue in RA, mainly in RA FLSs. Consistently, we found that XIAP was upregulated in the synovial tissues and cells of patients with RA; this was inversely proportional to miR-431-5p levels. Furthermore, we confirmed the binding between miR-431-5p and XIAP and effect of miR-431-5p on XIAP levels in RA FLSs. As expected, silencing XIAP expression partially reversed the effects of miR-431-5p inhibition on cell proliferation, apoptosis, and cell cycle progression in RA FLSs (Fig. 6). These results indicate that miR-431-5p exerts its protective roles by targeting XIAP in RA FLSs.

Our previous studies identified downregulation of miR-410-3p in RA FLSs and regulated cell proliferation, apoptosis, and cell cycle of RA FLSs [15]. Since miR-431-5p shared similar effects with miR-410-3p in RA FLSs, we wondered whether they also shared similar mechanism. Interestingly, miR-431-5p had no impact on YY1 levels, whereas overexpression of miR-410-3p significantly reduced XIAP levels in RA FLSs. These results suggest that some miRNAs, such as miR-410-3p and miR-431-5p, might exert synergetic effects through overlapping mechanisms in RA FLSs.

Our findings demonstrated the dysregulation of miR-431-5p and mechanism involved in the development of RA. However, this study still has some limitations. Few synovial tissues were used to assess miR-431-5p and XIAP levels; therefore, the results of comparison of miR-431-5p levels in synovial tissues might be biased. Due to difficulties of obtaining fresh synovial tissues, cells for all functional assays in this study were immortalized cell lines, rather than primary FLS, which might have some minor discrepancy. However, immortalized cell lines, such as HFLS-RA and MH7A cells, were commonly used as tool cells for biological process analysis in RA [34]. Furthermore, the therapeutic efficacy of miR-431-5p needs to be explored in animal models of RA.

In conclusion, this study shows that miR-431-5p was downregulated in synovial tissues and FLSs of patients with RA. Upregulation of miR-431-5p in RA FLSs suppressed cell proliferation and G0/G1-to-S phase transition and promoted apoptosis by targeting XIAP.

## Conclusions

We investigated the expression of miR-431-5p in synovial tissues and FLSs in RA and further explored the effect of miR-431-5p on cell proliferation, apoptosis, and cell cycle progression in RA FLSs. Here, we revealed that miR-431-5p was downregulated in synovial tissues and FLSs of patients with RA. Upregulation of miR-431-5p in RA FLSs suppressed cell proliferation and G0/G1-to-S phase transition and promoted apoptosis by targeting XIAP. Our findings therefore suggest miR-431-5p as a potential treatment target in RA.

## Supplementary information

**Additional file 1: Fig. S1.** Effects of miR-431-5p on YY1 levels in RA FLSs. A. qRT-PCR for the relative expression of YY1 in HFLS-RA cells transfected with miR-431-5p mimics or inhibitor. B, C. Western blotting to determine the levels of YY1 in HFLS-RA cells transfected with miR-431-5p mimics or inhibitor. Each experiment was performed independently in triplicates. ns no significance, compared with the NC groups.

**Additional file 2: Fig. S2.** Effects of miR-410-3p on XIAP levels in RA FLSs. A. qRT-PCR for the relative expression of XIAP in HFLS-RA cells transfected with miR-410-3p mimics. B, C. Western blotting to determine the levels of XIAP in HFLS-RA cells transfected with miR-410-3p mimics. Each experiment was performed independently in triplicates. * *P* < 0.05, **P < 0.01, compared with the NC groups.

**Additional file 3: Supplementary Table 1.** Clinical characteristics of patients with RA.

## Data Availability

The datasets used and analyzed during this study are available from the corresponding author on reasonable request.

## Abbreviations

RA: Rheumatoid arthritis
FLS: Fibroblast-like synoviocyte
miRNA: MicroRNA
XIAP: X-linked inhibitor of apoptosis protein
HFLS: Healthy human fibroblast-like synoviocyte
HFLS-RA: Human fibroblast-like synoviocyte from patients with rheumatoid arthritis
NC: Negative control
CCK-8: Cell counting kit-8
WT: Wild type
qRT-PCR: Quantitative real-time polymerase chain reaction
