# Supplementary figures and images for: miR-431-5p regulates cell proliferation and apoptosis in fibroblast-like synoviocytes in rheumatoid arthritis by targeting XIAP
Source: Arthritis Res Ther. 2020 Oct 6;22:231. doi: 10.1186/s13075-020-02328-3 (PMC7542379; doi:10.1186/s13075-020-02328-3)

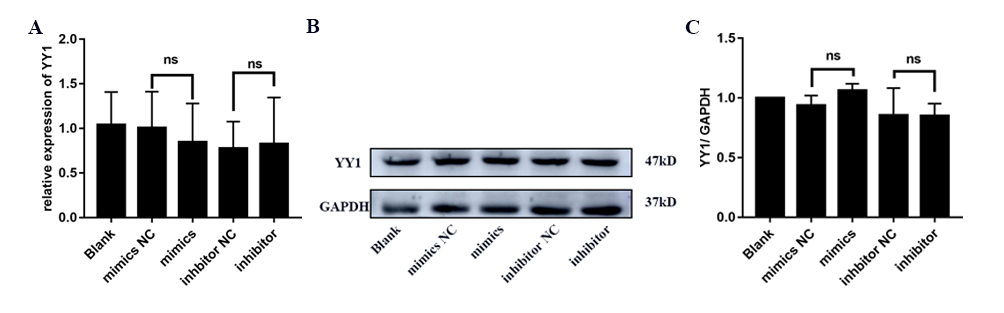

Supplement: Supplementary file 1 — Additional file 1: Fig. S1. Effects of miR-431-5p on YY1 levels in RA FLSs. A. qRT-PCR for the relative expression of YY1 in HFLS-RA cells transfected with miR-431-5p mimics or inhibitor. B, C. Western blotting to determine the levels of YY1 in HFLS-RA cells transfected with miR-431-5p mimics or inhibitor. Each experiment was performed independently in triplicates. ns no significance, compared with the NC groups. [file 13075_2020_2328_MOESM1_ESM.tif]

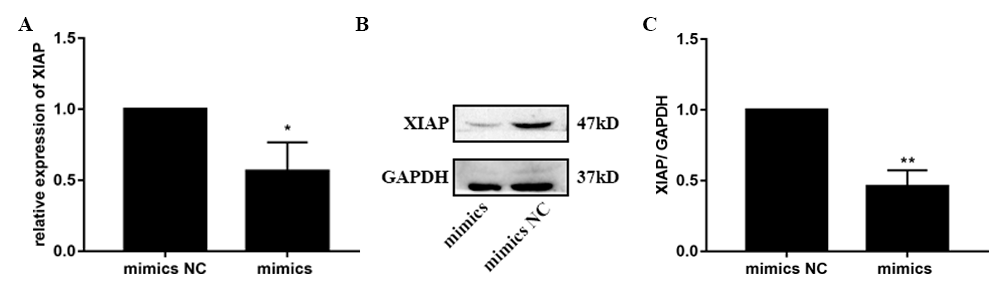

Supplement: Supplementary file 2 — Additional file 2: Fig. S2. Effects of miR-410-3p on XIAP levels in RA FLSs. A. qRT-PCR for the relative expression of XIAP in HFLS-RA cells transfected with miR-410-3p mimics. B, C. Western blotting to determine the levels of XIAP in HFLS-RA cells transfected with miR-410-3p mimics. Each experiment was performed independently in triplicates. * P < 0.05, **P < 0.01, compared with the NC groups. [file 13075_2020_2328_MOESM2_ESM.tif]
