# Supplementary material for: miR-431-5p regulates cell proliferation and apoptosis in fibroblast-like synoviocytes in rheumatoid arthritis by targeting XIAP
Source: Arthritis Res Ther. 2020 Oct 6;22:231. doi: 10.1186/s13075-020-02328-3 (PMC7542379; doi:10.1186/s13075-020-02328-3)
Supplement: Supplementary file 3 — Additional file 3: Supplementary Table 1. Clinical characteristics of patients with RA. [file 13075_2020_2328_MOESM3_ESM.docx]

Supplementary Table 1 Clinical characteristics of patients with RA

| No. | Age | Sex | Disease Duration (months) | RF | ACPA | DAS28-ESR scores |
| --- | --- | --- | --- | --- | --- | --- |
| 1 | 52 | Female | 16 | + | + | 5.56 |
| 2 | 49 | Female | 8 | + | + | 4.31 |
| 3 | 42 | Female | 24 | + | - | 4.93 |
| 4 | 55 | Female | 34 | - | + | 4.51 |
| 5 | 54 | Female | 14 | + | + | 3.97 |
| 6 | 39 | Female | 6 | - | + | 3.77 |
| 7 | 44 | Female | 12 | + | - | 3.28 |
| 8 | 48 | Female | 12 | + | + | 3.78 |

*RF* rheumatoid factor, *ACPA* anti-cyclic citrullinated peptide antibody, *DAS28-ESR* Disease Activity Score in 28 joints using erythrocyte sedimentation rate
